# Supplementary material for: Content-rich biological network constructed by mining PubMed abstracts
Source: BMC Bioinformatics. 2004 Oct 8;5:147. doi: 10.1186/1471-2105-5-147 (PMC528731; doi:10.1186/1471-2105-5-147)
Supplement: Additional File 2 — The original results of the above study (non-essential files are deleted to keep the file size under the limit set by BMC bioinformatics). [file 1471-2105-5-147-S2.bz2 › chilibotAdditionalFile2/dip20/607ID9786864E10989/html/LEP_LEPR.html]

 


 **LEP** and **LEPR** 
  
Found 5619 abstracts in PubMed, retrieved 20.  
 

 What does Google say? 
 PDF only 
| .edu only 

---

**Interactive relationship** (e.g. stimulation, inhibition, etc)

**Neutral relationship**- Significantly higher serum levels of TG and lower HDL C were observed in obese leptin  [ **LEPR** ]   [ **LEP** ]  resistant group, as compared with those in obese leptin  [ **LEPR** ]   [ **LEP** ]  sensitive group.  Ref: 12880566 Zhonghua Yu Fang Yi Xue Za Zhi, 2003
- CONCLUSIONS Together these data show that the effect of leptin  [ **LEPR** ]   [ **LEP** ]  on LV remodeling is not attributable to weight loss alone, indicating that leptin  [ **LEPR** ]   [ **LEP** ]  has antihypertrophic effects on the heart, either directly or through a leptin  [ **LEPR** ]   [ **LEP** ]  regulated neurohumoral pathway.  Ref: 12885755 CirculationCirculation, 2003
- Examination of relationships among plasma leptin  [ **LEPR** ]   [ **LEP** ] , proinflammatory cytokines, and stress related parameters revealed a significant positive correlation between the plasma leptin  [ **LEPR** ]   [ **LEP** ]  level and IL 1beta or IL 6.  Ref: 12880724 Burns, 2003
- Our results showed that the serum leptin  [ **LEPR** ]   [ **LEP** ]  levels of the bulimic patients were similar to those of the healthy controls, with a positive correlation between leptin  [ **LEPR** ]   [ **LEP** ]  and BMI.  Ref: 12880190 Eat Weight Disord, 2003
- RESULTS Serum level of leptin  [ **LEPR** ]   [ **LEP** ]  was 2.74 45.12 micro g L and 0.53 10.18 micro g L in obese and normally weighted children, respectively, with an average level of leptin  [ **LEPR** ]   [ **LEP** ]  log significantly higher in obese group than that in control group P <.  Ref: 12880566 Zhonghua Yu Fang Yi Xue Za Zhi, 2003
- Pretreatment of these cells with anti leptin  [ **LEPR** ]   [ **LEP** ]  receptor Ob R  [ **LEPR** ]  antibody, protein kinase C PKC inhibitors, calphostin C, and GF109203X, or the antioxidant N acetylcysteine NAC blocked the effects of leptin  [ **LEPR** ]   [ **LEP** ] .  Ref: 12882931 Diabetes, 2003

**Non-interactive relationship** (e.g. studied together, co-existance, homology, etc.)

- For testing whether chronic activation of the central melanocortin pathway can bypass the defective leptin  [ **LEPR** ]   [ **LEP** ]  signaling and normalize altered energy homeostasis in these rats, recombinant adeno associated virus encoding pro opiomelanocortin rAAV POMC or control vector was delivered bilaterally into the basal hypothalamus with coordinates targeting the arcuate nucleus.  Ref: 12882910 DiabetesDiabetes, 2003
- The levels of leptin  [ **LEPR** ]   [ **LEP** ]  a plasma protein synthesised in human adipose tissue are increased in obese subjects and significantly decrease after a major weight loss.  Ref: 12880185 Eat Weight Disord, 2003
- Leptin  [ **LEPR** ]   [ **LEP** ]  increases lipoprotein lipase secretion by macrophages involvement of oxidative stress and protein kinase C.  Ref: 12882931 Diabetes, 2003
- Disruption of Leptin  [ **LEPR** ]   [ **LEP** ]  Signaling Contributes to Cardiac Hypertrophy Independently of Body Weight in Mice.  Ref: 12885755 CirculationCirculation, 2003
- It has been shown that serum leptin  [ **LEPR** ]   [ **LEP** ]  levels correlate with weight and percentage body fat in normal and obese individuals, but the exact correlation between leptin  [ **LEPR** ]   [ **LEP** ]  and body weight in anorexic and bulimic patients has not yet been clarified.  Ref: 12880190 Eat Weight Disord, 2003
- In contrast, a three day period of GH administration had no effect on plasma leptin  [ **LEPR** ]   [ **LEP** ]  during LP or EL.  Ref: 12881203 Am J Physiol Regul Integr Comp PhysiolAm J Physiol Regul Integr Comp Physiol, 2003
- Univariate correlations revealed significant negative relationships between plasma adiponectin levels and BMI, percent body fat, visceral fat, subcutaneous abdominal fat, fasting leptin  [ **LEPR** ]   [ **LEP** ] , and fasting insulin and positive relationship with M all P &lt.  Ref: 12882866 Diabetes CareDiabetes Care, 2003
- Impact of dietary FA and energy restriction on plasma leptin  [ **LEPR** ]   [ **LEP** ]  and ob gene expression in mice.  Ref: 12880106 Lipids, 2003
- However, serum leptin  [ **LEPR** ]   [ **LEP** ]  concentrations of twins mean SEM 1.13 0.35 ng ml were significantly lower than of the singleton infants 4.27 0.63 ng ml p <.  Ref: 12880123 J Pediatr Endocrinol Metab, 2003
- A possible role of thyroid hormones in regulating adipose tissue metabolism in humans has been proposed, but it is not universally accepted and the relationship between thyroid function and leptin  [ **LEPR** ]   [ **LEP** ]  levels has not yet been clearly defined.  Ref: 12880185 Eat Weight Disord, 2003
- We studied serum leptin  [ **LEPR** ]   [ **LEP** ] , TSH, fT4 and fT3 levels in 38 obese patients 26 women and 12 men, before and 12 months after BPD.  Ref: 12880185 Eat Weight Disord, 2003
- To separate the direct contribution of leptin  [ **LEPR** ]   [ **LEP** ]  deficiency from mechanical effects of obesity, we induced weight loss in 6 to 8 month old ob ob mice either by leptin  [ **LEPR** ]   [ **LEP** ]  infusion or caloric restriction.  Ref: 12885755 CirculationCirculation, 2003
- Leptin  [ **LEPR** ]   [ **LEP** ] , principally secreted by the adipocytes, interacts with hypothalamic receptors, and is capable of activating diverse neuroendocrinal routes responsible for controlling the balance between energy intake and expenditure.  Ref: 12886336 An Sist Sanit Navar, 1999
- The absence of an association between plasma levels of androgens and prostate cancer risk in analytical studies, despite the strong indirect evidence of their tumour stimulating effects, may reflect the complex and mostly inverse associations of androgenicity to IGF I, insulin and leptin  [ **LEPR** ]   [ **LEP** ]  which are hormones that have also been implicated as risk factors for prostate cancer.  Ref: 12883384 Eur J Cancer Prev, 2003
- Fasting serum leptin  [ **LEPR** ]   [ **LEP** ] , insulin, and cholesterol levels were also significantly reduced by rAAV POMC treatment.  Ref: 12882910 DiabetesDiabetes, 2003
- The use of recombinant leptin  [ **LEPR** ]   [ **LEP** ]  in patients with obesity is mentioned.  Ref: 12884645 Przegl Lek, 2003
- The role of leptin  [ **LEPR** ]   [ **LEP** ]  in the etiopathogenesis of anorexia nervosa and bulimia.  Ref: 12880190 Eat Weight Disord, 2003
- The plasma leptin  [ **LEPR** ]   [ **LEP** ]  concentrations on ad libitum CH and MUFA diets did not differ significantly 24.3 2.1 and 34.7 6.7 ng mL, respectively whereas the saturated fat diet caused a lower concentration 13.9 1.9 ng mL.  Ref: 12880106 Lipids, 2003
- This decline mirrors that of plasma insulin, but is reciprocal to the profile of plasma growth hormone GH, suggesting that both hormones may regulate plasma leptin  [ **LEPR** ]   [ **LEP** ]  in periparturient dairy cows.  Ref: 12881203 Am J Physiol Regul Integr Comp PhysiolAm J Physiol Regul Integr Comp Physiol, 2003
- Leptin  [ **LEPR** ]   [ **LEP** ]  and body weight regulation .  Ref: 12886336 An Sist Sanit Navar, 1999
- OBJECTIVE To investigate the relationship between fasting serum levels of leptin  [ **LEPR** ]   [ **LEP** ] , glucose, insulin resistance, lipids in simple obese children.  Ref: 12880566 Zhonghua Yu Fang Yi Xue Za Zhi, 2003
- METHODS AND RESULTS We measured left ventricular LV wall thickness and LV mass with echocardiography in mice lacking leptin  [ **LEPR** ]   [ **LEP** ]  ob ob, n=15 or functional receptor db db, n=10 and controls at, and 6 months of age.  Ref: 12885755 CirculationCirculation, 2003
- Microarray mRNA analysis in adipose tissue revealed that leptin  [ **LEPR** ]   [ **LEP** ]  was the only up regulated gene affecting glucose uptake.  Ref: 12878760 J PhysiolJ Physiol, 2003
- Infusion of insulin 1 micro g kg body weight per hour caused a progressive rise in the plasma concentration of leptin  [ **LEPR** ]   [ **LEP** ]  that reached maximum levels at 24 h during both physiological states.  Ref: 12881203 Am J Physiol Regul Integr Comp PhysiolAm J Physiol Regul Integr Comp Physiol, 2003
- Leptin  [ **LEPR** ]   [ **LEP** ]  is a hormone that is released mainly by adipocytes.  Ref: 12884645 Przegl Lek, 2003
- Insulin infusion increased leptin  [ **LEPR** ]   [ **LEP** ]  mRNA in adipose tissue during LP but not during EL.  Ref: 12881203 Am J Physiol Regul Integr Comp PhysiolAm J Physiol Regul Integr Comp Physiol, 2003
- We previously showed that this energy deficit contributes to a decline in plasma leptin  [ **LEPR** ]   [ **LEP** ] .  Ref: 12881203 Am J Physiol Regul Integr Comp PhysiolAm J Physiol Regul Integr Comp Physiol, 2003
- Zucker fa fa rats with defective leptin  [ **LEPR** ]   [ **LEP** ]  receptors are obese, hyperphagic, and hyperinsulinemic.  Ref: 12882910 DiabetesDiabetes, 2003
- CONCLUSIONS The data suggest that plasma adiponectin does not change with age but levels are negatively associated with percent body fat, visceral fat, subcutaneous abdominal fat, insulin, and leptin  [ **LEPR** ]   [ **LEP** ]  levels in women.  Ref: 12882866 Diabetes CareDiabetes Care, 2003
- This study demonstrates that targeted POMC gene delivery in the hypothalamus suppresses food intake and weight gain and reduces visceral adiposity and hyperinsulinemia in leptin  [ **LEPR** ]   [ **LEP** ]  resistant obese Zucker rats.  Ref: 12882910 DiabetesDiabetes, 2003
- We investigated leptin  [ **LEPR** ]   [ **LEP** ]  levels in the serum of 58 female subjects aged 15 36 years 10 with bulimia nervosa BN.  Ref: 12880190 Eat Weight Disord, 2003
- Other factors, such as increased response of adipose tissue to beta adrenergic signals, probably contribute to the reduction of plasma leptin  [ **LEPR** ]   [ **LEP** ]  in early lactating dairy cows.  Ref: 12881203 Am J Physiol Regul Integr Comp PhysiolAm J Physiol Regul Integr Comp Physiol, 2003
- These results suggest that leptin  [ **LEPR** ]   [ **LEP** ]  is not only an adipostatic hormone but also a stress related hormone.  Ref: 12880122 J Pediatr Endocrinol Metab, 2003
- These results suggest that the plasma leptin  [ **LEPR** ]   [ **LEP** ]  level may have some relations to plasma proimflammatory cytokines in pathophysiologic responses to critical conditions of burn injury.  Ref: 12880724 Burns, 2003
- Of obese children, 83% were leptin  [ **LEPR** ]   [ **LEP** ]  resistant.  Ref: 12880566 Zhonghua Yu Fang Yi Xue Za Zhi, 2003
- Similar results were observed in leptin  [ **LEPR** ]   [ **LEP** ]  treated J774 macrophages.  Ref: 12882931 Diabetes, 2003
- Serum leptin  [ **LEPR** ]   [ **LEP** ]  levels in neonatal bacterial septicemia.  Ref: 12880122 J Pediatr Endocrinol Metab, 2003
- Plasma Adiponectin and Leptin  [ **LEPR** ]   [ **LEP** ]  Levels, Body Composition, and Glucose Utilization in Adult Women With Wide Ranges of Age and Obesity.  Ref: 12882866 Diabetes CareDiabetes Care, 2003
- Although there were positive correlations between serum leptin  [ **LEPR** ]   [ **LEP** ]  levels and serum CRP levels and IT ratio in the septicemic group at the start, there were no correlations between serum leptin  [ **LEPR** ]   [ **LEP** ]  levels and other hematological parameters.  Ref: 12880122 J Pediatr Endocrinol Metab, 2003
- Relationship between serum levels of leptin  [ **LEPR** ]   [ **LEP** ]  and glucose, lipids in simple obese children .  Ref: 12880566 Zhonghua Yu Fang Yi Xue Za Zhi, 2003
- OBJECTIVE The purpose of this study was to determine the relationships between plasma adiponectin and leptin  [ **LEPR** ]   [ **LEP** ]  levels, total and central obesity, and glucose utilization across the adult age span.  Ref: 12882866 Diabetes CareDiabetes Care, 2003
- Serum levels of TC, TG, LDL C and insulin were significantly higher in obese leptin  [ **LEPR** ]   [ **LEP** ]  resistant group than those in normally weighted control group, but no significant difference in them between obese leptin  [ **LEPR** ]   [ **LEP** ]  sensitive group and its normally weighted control group was observed.  Ref: 12880566 Zhonghua Yu Fang Yi Xue Za Zhi, 2003
- Effect of Insulin and Growth Hormone on Plasma Leptin  [ **LEPR** ]   [ **LEP** ]  in the Periparturient Dairy Cow.  Ref: 12881203 Am J Physiol Regul Integr Comp PhysiolAm J Physiol Regul Integr Comp Physiol, 2003
- We conclude that the increase in insulin resistance in response to intermittent hypoxia is dependent on the disruption of leptin  [ **LEPR** ]   [ **LEP** ]  pathways.  Ref: 12878760 J PhysiolJ Physiol, 2003
- Interestingly, no differences in plasma leptin  [ **LEPR** ]   [ **LEP** ]  levels between groups were seen in the energy restricted groups mean 8.0 1.0 ng mL.  Ref: 12880106 Lipids, 2003
- In these cells, leptin  [ **LEPR** ]   [ **LEP** ]  increased the membrane expression of conventional PKC isoforms and downregulation of endogenous PKC expression abolished the effects of leptin  [ **LEPR** ]   [ **LEP** ]  on macrophage LPL expression.  Ref: 12882931 Diabetes, 2003
- Leptin  [ **LEPR** ]   [ **LEP** ]  infusion completely reversed the increase in wall thickness with partial resolution of myocyte hypertrophy.  Ref: 12885755 CirculationCirculation, 2003
- Leptin  [ **LEPR** ]   [ **LEP** ]  resistance correlated significantly with the risk of metabolic syndrome and cardiovascular disease, indicating serum level of leptin  [ **LEPR** ]   [ **LEP** ]  could be used as an indicator in screening obese children at high risk.  Ref: 12880566 Zhonghua Yu Fang Yi Xue Za Zhi, 2003
- Compared with quintile 5, the odds of having an increased serum leptin  [ **LEPR** ]   [ **LEP** ]  level in quintiles, 3, and 4 were 2.26 95% confidence interval CI 1.54 to 3.3.  Ref: 12885988 Thorax, 2003
- METHODS Fasting serum levels of leptin  [ **LEPR** ]   [ **LEP** ]  and insulin Fins were measured by RIA in 42 obese and 42 normally weighted children matched on age, sex and height, and their total cholesterol TC, triglyceride TG, high density lipoprotein cholesterol HDL C were analyzed with enzymatic methods.  Ref: 12880566 Zhonghua Yu Fang Yi Xue Za Zhi, 2003
- 0.01, improvement in glucose tolerance without a change in serum insulin levels, and an increase in serum leptin  [ **LEPR** ]   [ **LEP** ]  levels in comparison with control 2.6 0.3 ng ml vs. 1.7 0.2 ng ml, p &lt.  Ref: 12878760 J PhysiolJ Physiol, 2003
- To further investigate this issue, we studied the interrelationships between androgenicity and insulin like growth factor I IGF I, insulin and leptin  [ **LEPR** ]   [ **LEP** ] .  Ref: 12883384 Eur J Cancer Prev, 2003
- Although bulimic patients have very bad nutritional behaviour, their leptin  [ **LEPR** ]   [ **LEP** ]  levels do not appear altered.  Ref: 12880190 Eat Weight Disord, 2003
- The aim of the study was to evaluate the possible correlations between leptin  [ **LEPR** ]   [ **LEP** ]  levels and the body mass index BMI in all five groups.  Ref: 12880190 Eat Weight Disord, 2003
- Overall, these data demonstrate that binding of leptin  [ **LEPR** ]   [ **LEP** ]  at the macrophage cell surface increases, through oxidative stress and PKC dependent pathways, LPL expression.  Ref: 12882931 Diabetes, 2003
- RESULTS Serum leptin  [ **LEPR** ]   [ **LEP** ]  levels changed along the FEV 1 gradient.  Ref: 12885988 Thorax, 2003
- Indices of free testosterone showed an inverse linear correlation with leptin  [ **LEPR** ]   [ **LEP** ]  P< ; 0.01, and a strong drop in the 5th quintile of BMI.  Ref: 12883384 Eur J Cancer Prev, 2003
- Plasma leptin  [ **LEPR** ]   [ **LEP** ]  levels in patients with burn injury a preliminary report.  Ref: 12880724 Burns, 2003
- Interrelationships between plasma testosterone, SHBG, IGF I, insulin and leptin  [ **LEPR** ]   [ **LEP** ]  in prostate cancer cases and controls.  Ref: 12883384 Eur J Cancer Prev, 2003
- The highest leptin  [ **LEPR** ]   [ **LEP** ]  levels were found in quintile 1 geometric mean GM 5.42.  Ref: 12885988 Thorax, 2003
- Therefore, insulin increases plasma leptin  [ **LEPR** ]   [ **LEP** ]  in LP by stimulating adipose tissue synthesis, but has only marginal effects in EL when cows are in negative energy balance.  Ref: 12881203 Am J Physiol Regul Integr Comp PhysiolAm J Physiol Regul Integr Comp Physiol, 2003
- CONCLUSION Twin pregnancy might be a factor affecting serum leptin  [ **LEPR** ]   [ **LEP** ]  concentrations in newborn infants.  Ref: 12880123 J Pediatr Endocrinol Metab, 2003
- The discovery of leptin  [ **LEPR** ]   [ **LEP** ] , besides causing a revolution in physiological knowledge about the regulation of body weight, awakes an interest in fat tissue as an endocrine organ and its active contribution to the establishment of energy deposits.  Ref: 12886336 An Sist Sanit Navar, 1999
- In contrast to adults, adiponectin levels in the fetus were unrelated to the degree of adiposity, blood insulin, or leptin  [ **LEPR** ]   [ **LEP** ]  in either control subjects or ODM.  Ref: 12882843 Diabetes CareDiabetes Care, 1999
- We utilized a mouse model to examine the effects of intermittent hypoxia on insulin resistance in lean C57BL 6J mice and leptin  [ **LEPR** ]   [ **LEP** ]  deficient obese C57BL 6J  **Lep**  ob mice.  Ref: 12878760 J PhysiolJ Physiol, 1999
- Like other cytokines, leptin  [ **LEPR** ]   [ **LEP** ]  is also able to play a role in the complex regulation of diverse metabolic aspects.  Ref: 12886336 An Sist Sanit Navar, 1999
- This increase in insulin secretion after 5 days of intermittent hypoxia was completely abolished by prior leptin  [ **LEPR** ]   [ **LEP** ]  infusion.  Ref: 12878760 J PhysiolJ Physiol, 1999
- Much evidence suggests that leptin  [ **LEPR** ]   [ **LEP** ]  plays the same roles in human beings.  Ref: 12884645 Przegl Lek, 2003
- The aim of the present study was to elucidate whether the qualitative composition of dietary fat influences plasma leptin  [ **LEPR** ]   [ **LEP** ]  and adipose tissue ob gene expression differentially.  Ref: 12880106 Lipids, 2003
- Serum leptin  [ **LEPR** ]   [ **LEP** ]  levels in twins and singleton newborns.  Ref: 12880123 J Pediatr Endocrinol Metab, 2003
- One of the roles of leptin  [ **LEPR** ]   [ **LEP** ]  in animals is regulation of the energy balance by decreasing food intake and increasing energy expenditure.  Ref: 12884645 Przegl Lek, 2003
- Impaired lung function and serum leptin  [ **LEPR** ]   [ **LEP** ]  in men and women with normal body weight a population based study.  Ref: 12885988 Thorax, 2003
- CONCLUSION Individuals with impaired lung function have raised serum leptin  [ **LEPR** ]   [ **LEP** ]  levels.  Ref: 12885988 Thorax, 2003
- Body size at ages 2 4 months was correlated with levels of serum leptin  [ **LEPR** ]   [ **LEP** ]  in both males and females, and with levels of IGF I and thyroid hormone in females only.  Ref: 12882350 Aging Cell, 2002
- The change in individual plasma leptin  [ **LEPR** ]   [ **LEP** ]  levels did not show similar pattern in all these patients.  Ref: 12880724 Burns, 2003
- Disruption of leptin  [ **LEPR** ]   [ **LEP** ]  signaling may represent a novel mechanism in LVH and related cardiovascular disorders.  Ref: 12885755 CirculationCirculation, 2003
- Treatment of human macrophages with leptin  [ **LEPR** ]   [ **LEP** ]  1 10 nmol l increased LPL expression, at both the mRNA and protein levels.  Ref: 12882931 Diabetes, 2003
- Within a prospective cohort study, we measured testosterone, sex hormone binding globulin SHBG and IGF I, IGF binding protein IGFBP 1, IGFBP 3, insulin and leptin  [ **LEPR** ]   [ **LEP** ] , in plasma from 149 cases and 298 controls.  Ref: 12883384 Eur J Cancer Prev, 2003
- Our data suggest that, although significantly reduced, serum leptin  [ **LEPR** ]   [ **LEP** ]  levels in fasting anorexic patients are non linearly related to body weight BMI.  Ref: 12880190 Eat Weight Disord, 2003
- Serum leptin  [ **LEPR** ]   [ **LEP** ]  was significantly p< ; 0.001 reduced in the anorexic patients because of the dramatic decrease in adipose mass caused by the nutritional defect, as is further supported by the significantly p< ; 0.001 low level of transferrinemia.  Ref: 12880190 Eat Weight Disord, 2003
- A study was undertaken to determine whether leptin  [ **LEPR** ]   [ **LEP** ] , a hormone that can promote atherothrombosis, is raised in individuals with impaired lung function.  Ref: 12885988 Thorax, 2003
- Two weeks after treatment, serum leptin  [ **LEPR** ]   [ **LEP** ]  levels in newborns with septicemia had decreased and were similar to those of controls.  Ref: 12880122 J Pediatr Endocrinol Metab, 2003
- Evaluation of serum leptin  [ **LEPR** ]   [ **LEP** ]  levels and thyroid function in morbidly obese patients treated with bariatric surgery.  Ref: 12880185 Eat Weight Disord, 2003
- ... testosterone and SHBG correlated inversely with IGF I, IGFBP 3, insulin, leptin  [ **LEPR** ]   [ **LEP** ]  and body mass index BMI.  Ref: 12883384 Eur J Cancer Prev, 2003
- The principal aim of this study was to evaluate serum leptin  [ **LEPR** ]   [ **LEP** ]  concentrations and to analyze the interaction between serum leptin  [ **LEPR** ]   [ **LEP** ]  levels and C reactive protein CRP levels, hematological parameters before and after antimicrobial therapy in neonates with bacterial septicemia.  Ref: 12880122 J Pediatr Endocrinol Metab, 2003
- At steady states, the absolute increase in plasma leptin  [ **LEPR** ]   [ **LEP** ]  was greater in LP than in EL cows 2.4 ng ml vs 0.4 ng ml.  Ref: 12881203 Am J Physiol Regul Integr Comp PhysiolAm J Physiol Regul Integr Comp Physiol, 2003
- METHODS Data from non obese participants in the Third National Health, Nutrition, and Examination Survey n=2808 were analysed to determine the relationship between circulating leptin  [ **LEPR** ]   [ **LEP** ]  levels and forced expiratory volume in 1 second FEV 1 values divided into quintiles quintile 1, FEV 1 predicted <.  Ref: 12885988 Thorax, 2003
- In order to find out whether multiple pregnancy is a factor affecting serum leptin  [ **LEPR** ]   [ **LEP** ]  levels, we compared neonatal serum leptin  [ **LEPR** ]   [ **LEP** ]  concentrations of twins and singleton newborns who were all appropriate for gestational age AGA.  Ref: 12880123 J Pediatr Endocrinol Metab, 2003
- BACKGROUND AND OBJECTIVE Leptin  [ **LEPR** ]   [ **LEP** ]  is produced by the human placenta besides adipose tissue and is suggested to be related to fetal growth.  Ref: 12880123 J Pediatr Endocrinol Metab, 2003
- This brief overview focuses on the history of leptin  [ **LEPR** ]   [ **LEP** ]  discovery, regulation of circulating leptin  [ **LEPR** ]   [ **LEP** ]  levels, and physiological role scientifically proved and being under research.  Ref: 12884645 Przegl Lek, 2003
- Thus, short term administration of a diet rich in SAFA suppresses circulating leptin  [ **LEPR** ]   [ **LEP** ]  levels without altering the adipose tissue ob gene expression.  Ref: 12880106 Lipids, 2003
- There was a significant post surgical decrease in BMI and circulating leptin  [ **LEPR** ]   [ **LEP** ]  levels in all of the treated subjects, but thyroid function did not seem to be affected TSH and fT4 levels were unchanged.  Ref: 12880185 Eat Weight Disord, 2003
- During lactation, mammary epithelial cells expressed leptin  [ **LEPR** ]   [ **LEP** ]  mRNA, but insulin did not increase milk leptin  [ **LEPR** ]   [ **LEP** ]  output.  Ref: 12881203 Am J Physiol Regul Integr Comp PhysiolAm J Physiol Regul Integr Comp Physiol, 2003
- The purpose of this study was to investigate the relationship between the plasma leptin  [ **LEPR** ]   [ **LEP** ]  level and clinical parameters in patients with burn injury.  Ref: 12880724 Burns, 2003
- This paper reviews the role of leptin  [ **LEPR** ]   [ **LEP** ]  in the regulation of body weight, as well as different aspects related to obesity.  Ref: 12886336 An Sist Sanit Navar, 1999
- However, leptin  [ **LEPR** ]   [ **LEP** ]  levels remained within the normal range, except in a patient Case 1 complicated with severe hypovolemic shock.  Ref: 12880724 Burns, 2003
- To gain further insight into the role of leptin  [ **LEPR** ]   [ **LEP** ]  in atherogenesis associated with diabetes, we investigated in the present study the role of this hormone in the regulation of macrophage lipoprotein lipase LPL, a proatherogenic cytokine overexpressed in patients with type 2 diabetes.  Ref: 12882931 Diabetes, 2003
- Leptin  [ **LEPR** ]   [ **LEP** ]  may play a role in the pathogenesis of cardiovascular morbidity and mortality related to impaired lung function.  Ref: 12885988 Thorax, 2003
- In leptin  [ **LEPR** ]   [ **LEP** ]  treated J774 cells, enhanced LPL synthetic rate and increased binding of nuclear proteins to the activated protein 1 AP 1 consensus sequence of the LPL gene promoter were also observed.  Ref: 12882931 Diabetes, 2003
- Since the discovery that the placenta, gastric mucous or the star shaped cells of the liver Kupffer cells, are places that produce leptin  [ **LEPR** ]   [ **LEP** ] , a great range of functions have been attributed to this hormone.  Ref: 12886336 An Sist Sanit Navar, 1999
- This indicates that saturated fat may alter protein handling by adipose tissue or the whole body clearance of leptin  [ **LEPR** ]   [ **LEP** ] .  Ref: 12880106 Lipids, 2003
- The mean concentration of serum leptin  [ **LEPR** ]   [ **LEP** ] , CRP levels, and immature total neutrophil IT ratio in newborns with septicemia were significantly higher than those of controls at the start.  Ref: 12880122 J Pediatr Endocrinol Metab, 2003
- The CLA isomers and fenofibrate altered mRNA levels for several proteins involved in lipid metabolism, but the most striking difference was the reduction of mRNA for leptin  [ **LEPR** ]   [ **LEP** ]  and adiponectin in the t10, c12 CLA group.  Ref: 12880104 Lipids, 2003
- Dysregulation of leptin  [ **LEPR** ]   [ **LEP** ] , a neurohormone essential to energy homeostasis, is implicated in the pathogenesis of obesity.  Ref: 12885755 CirculationCirculation, 2003
- Serum samples were taken at 24 hours following delivery and leptin  [ **LEPR** ]   [ **LEP** ]  concentrations were measured.  Ref: 12880123 J Pediatr Endocrinol Metab, 2003
- Plasma levels were monitored for leptin  [ **LEPR** ]   [ **LEP** ] , proinflammatory cytokines interleukin IL 1beta, IL 6, tumor necrosis factor alpha TNF alpha, stress related parameters adrenocorticotropic hormone ACTH, cortisol, and C reactive protein CRP.  Ref: 12880724 Burns, 2003
- Serum level of leptin  [ **LEPR** ]   [ **LEP** ]  was positively correlated with BMI, WHR and percentage of body fat.  Ref: 12880566 Zhonghua Yu Fang Yi Xue Za Zhi, 2003
- Recent data suggest that plasma leptin  [ **LEPR** ]   [ **LEP** ]  may represent a cardiovascular risk factor in diabetic patients.  Ref: 12882931 Diabetes, 2003
- CONCLUSIONS A big difference in serum level of leptin  [ **LEPR** ]   [ **LEP** ]  between obese and normally weighted children was found, suggesting most obese children were resistant to endogenous leptin  [ **LEPR** ]   [ **LEP** ] .  Ref: 12880566 Zhonghua Yu Fang Yi Xue Za Zhi, 2003
- Leptin  [ **LEPR** ]   [ **LEP** ]  is an adipocyte derived signal factor 167 amino acid protein encoded by the ob gene in chromosome 7q31 that regulates eating behaviour via central neuroendocrine mechanisms.  Ref: 12880190 Eat Weight Disord, 2003
- Because leptin  [ **LEPR** ]   [ **LEP** ]  has cardiovascular bioactivity, we hypothesized that disruption of leptin  [ **LEPR** ]   [ **LEP** ]  signaling mediates the development of obesity associated LVH.  Ref: 12885755 CirculationCirculation, 2003
- Besides, leptin  [ **LEPR** ]   [ **LEP** ]  participates es in the immune and inflammatory responses, in reproduction, in processes related to angiogenesis, control of arterial pressure, lipolysis, etc.  Ref: 12886336 An Sist Sanit Navar, 1999
